# Prise en charge des larges fistules bilio-kystiques lors de la chirurgie conservatrice du kyste hydatique du foie

**DOI:** 10.11604/pamj.2021.38.195.27098

**Published:** 2021-02-22

**Authors:** Aymen Trigui, Haitham Rejab, Jihene Krichene, Ahmed Tlili, Jihene Trabelsi, Ahmed Kachoua, Salah Boujelbene, Rafik Mzali

**Affiliations:** 1 Department of General and Digestive Surgery, Faculty of Medicine, University of Sfax, Habib Bourguiba Hospital, Sfax, Tunisia,; 2 Department of General and Digestive Surgery, Faculty of Medicine, University of Sfax, Ibn El Jazzar Hospital, Kairouan, Tunisia,; 3 Department of General and Digestive Surgery, Faculty of Medicine, University of Sfax, Mohamed Ben Sassi Hospital, Gabes, Tunisia,; 4 Department of Epidemiology, Faculty of Medicine, University of Sfax, Hedi Cheker Hospital, Sfax, Tunisia

**Keywords:** Kyste hydatique, foie, fistule biliaire, Hydatid cyst, liver, biliary fistula

## Abstract

**Introduction:**

le traitement des larges fistules kysto-biliaires ne fait pas l'unanimité des auteurs en l'absence de consensus ou de niveau de preuve élevé. Les méthodes chirurgicales sont controversées entre traitement radical permettant de suturer la fistule en zone saine et le traitement conservateur qui pose des problèmes de réparation. Le but de cette étude est de comparer les différentes techniques chirurgicales du traitement conservateur des larges fistules kysto-biliaires.

**Méthodes:**

étude rétrospective de 54 fistules larges, menée au service de chirurgie générale du CHU Habib Bourguiba à Sfax durant 9 ans (2010 - 2018).

**Résultats:**

il s'agit de 44 patients. L'échographie abdominale a permis de suspecter une ouverture dans les voies biliaires dans 18 cas (47,4%) alors que la tomodensitométrie (TDM), a suspecté une ouverture chez 28 patients (68.3%). Le traitement des fistules était réalisé par la technique du DITFO dans 18 cas (33,3%), de PERDROMO dans 11 cas (20,4%) et le drainage bipolaire dans 25 cas. Le taux de morbidité chirurgicale spécifique était de 31,5% dominé par une fistule biliaire post opératoire dans 18,5% des cas. La technique de DITFO était associée à un séjour hospitalier le plus court (p=0,028) et une morbidité plus basse (22,2%) sans différence statistiquement significative.

**Conclusion:**

la technique de DITFO représente la technique de choix dans le traitement des fistules kysto-biliaires (FKB), elle a une morbidité moins élevée et le séjour hospitalier le plus court.

## Abstract (en)

**Introduction:**

the treatment of large biliocystic fistulas is not unanimous among authors in the absence of consensus or a high level of evidence. There is a controversy over the use of a radical approach which allows the fistula to be sutured in a healthy area or conservative treatment that poses repair issues. The purpose of this study is to compare different conservative techniques to treat large biliocystic fistulas.

**Methods:**

we conducted a retrospective study of 54 patients with large fistulas in the Department of General Surgery at the Habib Bourguiba University Hospital in Sfax over a period of 9 years (2010 - 2018).

**Results:**

fourty-four patients were enrolled in the study. Abdominal ultrasound suggested opening of the bile ducts in 18 cases (47.4%) while computed tomography (CT) scan suggested opening in 28 patients (68.3%). The treatment of fistulas was based on DITFO (internal trans-fistulary drainage) in 18 cases (33.3%), cystobiliary disconnection (PERDROMO) in 11 cases (20.4%) and bipolar drainage in 25 cases. Specific surgical morbidity rate was 31.5% and it was dominated by postoperative biliary fistula in 18.5% of cases. DITFO technique was associated with shorter hospital stay (p=0.028) and lower morbidity rates (22.2%) with no statistically significant difference.

**Conclusion:**

DITFO technique is the gold standard technique in the treatment of biliocystic fistula because it is associated with lower morbidity rates and the shortest hospital stay.

## Introduction

Le KHF continue jusqu´à aujourd´hui à être un problème de santé publique en Tunisie non seulement en raison de sa fréquence mais également à cause des difficultés thérapeutiques qu´il peut engendrer notamment en cas de complication telle que l´ouverture du kyste dans les voies biliaires. En effet une large fistule kysto-biliaire est un tournant évolutif dans la maladie hydatique. Les méthodes chirurgicales sont controversées entre traitement radical permettant de suturer la fistule en zone saine et le traitement conservateur qui pose des problèmes de réparation. Le but de cette étude rétrospective est de comparer les différentes techniques chirurgicales du traitement conservateur des larges fistules kysto-biliaires.

## Méthodes

Nous avons mené une étude rétrospective au service de chirurgie générale au CHU Habib Bourguiba à Sfax durant 9 ans du 01/01/2010 au 31/07/2018. Nous avons inclus les larges fistules kysto-biliaires dont le diamètre est supérieur ou égal à 5mm. Le caractère large de la fistule (5mm) a été attesté par le passage de l´extrémité de la pince à calcul à travers la fistule ou le passage de la sonde de Nélaton CH16. Pour le scanner et l´échographie nous avons retenu comme signes directs la visualisation directe de la fistule, comme signes indirects la dilatation de l´arbre biliaire et/ou visualisation du matériel hydatique dans la voie biliaire principal. Nous avons retenu comme complications spécifiques aux fistules larges: la fistule biliaire est définie par l´écoulement de bile clair à travers le drainage quel que soit la date de déclaration en post opératoire et quel que soit la quantité, la rétention purulente définie par la survenue d´infection de la cavité résiduelle, la suppuration de la cavité résiduelle définie par l´infection de la cavité résiduelle avec drainage en place et la rétention cavitaire biliaire définie par une rétention faite de bile stérile.

La saisie et l´analyse des données a été faite par le logiciel SPSS. 18.0. La description des variables qualitatives a été faite par le calcul des effectifs observés et des fréquences relatives (pourcentages). Pour les variables quantitatives, nous avons calculé la moyenne arithmétique et l´écart-type pour les variables dont la distribution est gaussienne et la médiane et les extrêmes dans le cas contraire. L´étude des caractéristiques des fistules, de leur traitement, de leurs complications et des facteurs associés aux complications a été faite en considérant les fistules comme unités d´étude.

## Résultats

Durant la période d´étude, parmi les 356 patients qui ont été opérés pour KHF, 44 (12,3%) avaient un KHF ouvert dans les voies biliaires. La moyenne d´âge de nos patients était de 43 ans avec des extrêmes allants de 16 à 77 ans. Il s´agissait de 3 hommes et 21 femmes avec un sex-ratio de l´ordre de 1,09. Tous nos patients étaient symptomatiques. La douleur était le maitre symptôme retrouvé dans 100% des cas. Elle était isolée dans 13 cas (29,5%). La fièvre était présente dans 25 cas (56,8%), elle était associée à une angiocholite aiguë dans 18 cas soit 72% des cas. L´ictère était présent dans 24 cas (54,5%). Il était associé à une angiocholite aiguë dans 18 cas soit 76% des cas. L´examen abdominal a révélé une douleur à la palpation chez 35 patients (94,6%) et une défense dans 2 autres cas (5,4%). Cette défense a été en rapport avec un tableau d´angiocholite aiguë. Nous avons noté la présence d´une masse abdominale chez 4 patients (9,1% des cas). L´hyperleucocytose a été présente dans 26 cas (59,1%). Le taux moyen des leucocytes était de 13570 éléments/mm^3^ avec un écart type de 7470 éléments/mm^3^. La cholestase biologique était constatée chez 35 patients soit 79,5%. La cytolyse hépatique était observée chez 34 patients dont 2 étaient isolées. Parmi les 9 patients qui n´avaient pas de cholestase, 4 patients avaient un bilan hépatique strictement normal (9%), à peu près un patient sur 10 avait un bilan hépatique strictement normal.

L´échographie abdominale a été faite dans 38 cas (86,4%). La majorité des kystes étaient type 3 et 4 soient respectivement 52% et 39,5% (38 échographies). L´échographie n´a permis de détecter des signes directs et indirects d´ouverture du kyste dans les voies biliaires que dans 20 cas parmi 38 soit 55,30%. La fistule était visible, soit un signe direct, à l´échographie dans 8 cas (21,05%). Elle était segmentaire dans 7 cas (87,5%) et sectorielle dans 1 cas (12,5%). La dilatation des voies biliaires a été détectée chez 20 patients. Elle était segmentaire dans 4 cas (20%), intéressant les VBIHG dans 2 cas. Parmi les dilatations segmentaires, un patient avait une fistule visible à l´échographie qui était en faveur d´une ouverture du kyste dans les voies biliaires. Pour les 3 autres, les dilatations segmentaires étaient associées à des kystes hilaires qui évoquent plutôt une compression qu´une ouverture dans les voies biliaires.

La TDM a été réalisée chez 41 patients (93,1%). L´ouverture du KHF dans les voies biliaires a été suspectée par les signes directs et indirects chez 28 patients soit 68.3% des cas. Parmi ces cas, 4 patients n´avaient ni ictère ni fièvre et 7 n´avaient pas de cholestase biologique. La fistule était visible à la TDM dans 19 cas (46,3%). Elle était segmentaire dans 9 cas (47,5%) et sectorielle dans 8 cas (41,1%). La TDM a permis de calculer la taille de 8 fistules dont la taille variait de 5 à 26mm avec une médiane de 8,5mm. Une dilatation des voies biliaires (28 cas, 68%) intéressait l´arbre biliaire dans 24 cas (85,7%). La dilatation segmentaire (N=4) était associée dans 2 cas à la visualisation directe de la fistule évoquant une ouverture du kyste dans les voies biliaires. Pour les deux autres cas (dilatation isolée), elle évoquait plutôt une compression biliaire plutôt qu´une ouverture dans les voies biliaires. Le matériel hydatique était visualisé dans la VBP dans 13 cas (31,7%). Une association avec la dilatation des VB a été observée dans tous les cas.

Tous nos patients ont été opérés par laparotomie. Trente-deux patients (72,1%) avaient un seul kyste et 5 avaient une polykystose soient 11,3%. La localisation la plus fréquente des kystes était respectivement: 15 kystes au segment IV, 12 kystes au segment VII, 14 kystes au segment VIII. L´exploration peropératoire des kystes a montré que 14 kystes avaient une taille supérieure à 10 cm (31,8%). La médiane de la taille des kystes a été de 80mm avec des extrêmes de 25mm à 220mm. La résection du dôme saillant a été faite dans 40 cas (90,9%). Pour les autres (4 cas), l´abord du kyste a été fait par une cholédocotomie dans 3 cas (6,8%) et par une hépatotomie antérieure, kystotomie et évacuation du kyste dans 1 cas. Dans notre série, les 44 kystes étudiés étaient compliqués de 54 fistules larges. Les fistules n´étaient visibles et identifiables macroscopiquement que dans 17 cas soient 31,4%. Elles étaient découvertes suites à la CPO dans 23 cas soient 42,5% et par le recours à l´épreuve au bleu de méthylène dans 14 cas soient 25,9%. L´identification macroscopique n´a été faite que dans 35% pour les liquides bilio ou bilio purulent.

**Figure 1:**
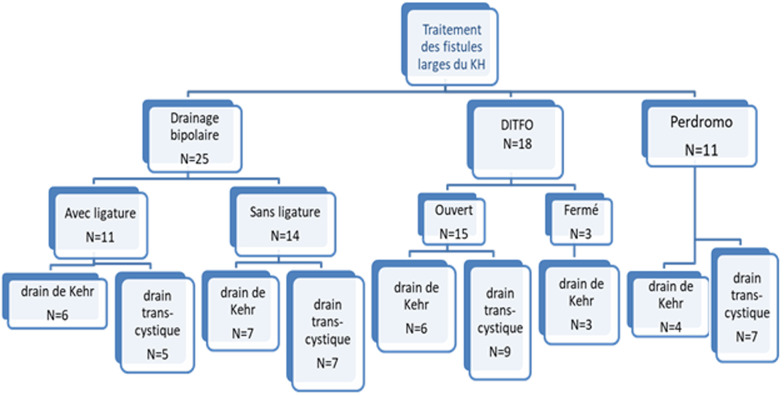
schéma récapitulatif du traitement des larges fistules

La localisation des fistules aux niveaux des canaux segmentaires était la plus fréquente (31 fistules segmentaires; 57,4%). Presque la moitié des fistules étaient opérés par un drainage bipolaire qui a été réalisé dans 25 cas soit 46,3% des cas. Le drainage des voies biliaires était par drain de kehr dans 13 cas 52% et par un drain trans cystique dans 12 cas soient 48%. Le DITFO était utilisé pour le traitement de 18 fistules larges soit 33,3%. Il était ouvert pour 15 fistules (27,8%) et fermé pour 3 fistules (5,6%). Le drainage des voies biliaire a été fait par drain de Kehr dans 9 cas (50%) et le drain trans cystique dans les autres cas (50%). Onze fistules étaient traitées selon PERDROMO dont la sonde d´intubation était le drain de kher dans 2 cas (18%). Et la sonde de Nélaton dans les autres cas. Le drainage des voies biliaires était par drain de Kehr dans 4 cas (36,4%) et par un drain trans cystique dans 7 cas (63,6%) (Tableau 1, Figure 1).

**Tableau 1:** traitement des fistules

Méthode chirurgicale		Effectif	Fréquence (%)
DITFO		18	33,3
Perdromo		11	20,4
Drainage bipolaire		25	46,3
	Drainage bipolaire avec Ligature	11	20,4%
	Drainage bipolaire avec respect de la fistule	14	25,9%
Total		54	100

DITFO : drainage interne transfistulo-oddien

Les suites étaient simples pour 35 fistules (64,8%) et compliquées pour 19 fistules (35,2%). La complication la plus fréquente était la fistule biliaire dans 10 cas (18,5%) avec un délai moyen de 3,22 jours (écart type 2,1 jours). La rétention purulente a été constatée chez 5 patients (9,3%), le cholépéritoine dans 3 cas (5,6%) et un obstacle résiduel sur la VBP a été rencontrée chez un patient traité par DITFO (1,9%) (Tableau 2). Il n´y avait pas d´association statistiquement significative entre les complications spécifiques et les facteurs cliniques, les critères biologiques et les caractéristiques des kystes (Tableau 3).

**Tableau 2:** récapitulation des complications par fistules larges du KH opérées pour chaque méthode thérapeutique

	DITFO	PERDRMO	Drainage bipolaire
	**N=18 (%)**	**N=11 (%)**	**N=25 (%)**
Morbidité globale (N=19)	5(27,8)	4(36,4)	10(40)
Morbidité spécifique (N=17)	4(22,2)	4(36,4)	9(36)
Fistule biliaire (N=10)	1(5,9)	2(18,2)	7(28)
Rétention purulente (N=5)	3(16,7)	1(9,1)	1(4)
Cholé-péritoine (N=3)	0	1(9,1)	2(8)
Obstacle résiduel sur la VBP (N=1)	1(5,6)	0	0

DITFO: drainage interne transfistulo-oddien

**Tableau 3:** étude des facteurs associés aux complications spécifiques

Critère qualitatif		N (%)	p
Sexe	Féminin (N=24)	7 (29,1)	0,74
	Masculin (N=30)	10 (33,3)	
ASA I	Oui (N=46)	16 (34,8)	0,41
	Non (N=8)	1 (12,5)	
Opéré pour KHF	Oui (N=3)	0	0,54
	Non (N=51)	17 (31,5)	
Angiocholite	Oui (N=20)	6 (30)	0,85
	Non (N=34)	11 (32,4)	
Ictère	Oui (N=26)	9 (34,6)	0,63
	Non (N=28)	8 (28,6)	
Cholestase	Oui (N=41)	14 (34,1)	0,51
	Non (N=13)	3 (23,1)	
Groupe actif	Oui (N=28)	11 (39,3)	0,2
	Non (N=26)	6 (23,1)	
Groupe transitionnel	Oui (N=14)	2 (14,3)	0,18
	Non (N=40)	15 (37,5)	
Groupe inactif	Oui (N=12)	4 (33,3)	1
	Non (N=42)	13 (31)	
Dôme hépatique	Oui (N=17)	6 (35,3)	0,68
	Non (N=37)	11 (29,7)	
Kyste périhilaire	Oui (N=23)	7 (30,4)	0,88
	Non (N=31)	10 (32,3)	
Localisation bilobaire	Oui (N=4)	0	0,29
	Non (N=50)	17 (34)	
Kyste du foie droit	Oui (N=26)	9 (34,6)	0,63
	Non (N=28)	8 (28,6)	
Kyste du foie gauche	Oui (N=24)	8 (33,3)	0,79
	Non (N=30)	9 (30)	

Il n´y avait aucune association entre la technique chirurgicale et la survenue de complications spécifiques (Tableau 4). Le séjour médian d´hospitalier pour la technique de PERDROMO était de 22 jours, alors que celui de la technique du DITFO était de 11 jours (Tableau 5). La durée d´hospitalisation des sujets ayant eu la technique PERDROMO était significativement plus importante par rapport aux autres techniques. Par contre, celle des sujets ayant eu la technique DITFO a été la moins importante.

**Tableau 4:** étude de l'association entre la morbidité spécifique et les techniques chirurgicales

Critère qualitatif	Technique	N (%)	p
Morbidité spécifique	DITFO (N=18)	4 (22,2)	0,3
	Perdromo (N=36)	4 (36,4)	0,72
	DB (N=25)	9 (36)	0,48
Rétention purulente	DITFO (N=18)	3 (16,7)	0,31
	Perdromo (N=36)	1(9,1)	1
	DB (N=25)	1 (4)	0,35
Obstacle résiduel	DITFO (N=18)	1 (5,6)	0,33
	Perdromo (N=36)	0	
	DB (N=25)	0	
Cholépéritoine	DITFO (N=18)	0	0,54
	Perdromo (N=36)	1 (9,1)	0,5
	DB (N=25)	2 (8)	0,59
Fistule biliaire	DITFO (N=18)	1 (5,9)	0,13
	Perdromo (N=36)	2(18,2)	0,97
	DB (N=25)	7 (28)	0,15

DITFO: drainage interne transfistulo-oddien; DB: drainage bipolaire

**Tableau 5:** la durée du séjour hospitalier en fonction des méthodes chirurgicales

		PERDROMO	DITFO	DB	DB avec ligature	DB sans ligature
Durée d’hospitalisation (j)	Médiane	22	11	14	12	15
Médiane (extrêmes)	Extrêmes	13-55	8-66	8-88	8-88	9-25
	p	0,004	0,028	0,97	0,34	0,56

DITFO : drainage interne transfistulo-oddien; DB: drainage bipolaire

## Discussion

L´ouverture du KHF dans les voies biliaires est la complication la plus redoutable. Elle varie selon les séries de 3,9% à 26,6%. Malgré l´apport du traitement médical, des traitements percutanés et endoscopiques, le traitement du KHF largement ouvert dans les voies biliaires reste essentiellement chirurgical. La chirurgie a plusieurs cibles: le parasite, le périkyste (avec le problème de la cavité résiduelle) et la fistules kysto-biliaire. La cavité résiduelle peut être traitée par 2 méthodes chirurgicales: la chirurgie conservatrice (CC) et la chirurgie radicale (CR). Le traitement radical permettrait de diminuer le risque de récidive et le risque de fistule biliaire externe puisque son traitement se fait en parenchyme hépatique sain; cependant, la CR ne peut être proposé pour des patients présentant une complication à type d´angiocholite aiguë et exposerait à un risque hémorragique plus accrue comparé à la CC, d´autant plus qu´elle nécessite une expertise en termes de chirurgie hépatique. Une méta-analyse récente publiée en 2017 [1] qui a comparé la chirurgie radicale à la chirurgie conservatrice du KHF a conclu que la chirurgie radicale était supérieure à la chirurgie conservatrice en termes de complications post opératoires et de récidive hydatique. En revanche, elle a montré une augmentation significative du temps opératoire et de la mortalité (non significative: 3.4% vs 3.2%).

En cas de traitement conservateur, le traitement d´une large fistule est différent des fistulettes. Différentes procédures sont décrites dans la littérature: la DITFO assure le drainage de la cavité résiduelle par voie naturelle unipolaire dans les voies biliaires à travers une large fistule kysto-biliaire qu´on respecte [2]. La technique PERDROMO [3] est fondé sur la déconnexion kysto-biliaire. La fistule kysto-biliaire étant intubée par un cathéter dont le diamètre est adapté à celui de la fistule. Ce cathéter a un court trajet intra cavitaire ne dépassant pas les 2cm et un trajet trans hépatique en tissu sain d´au moins de 3cm et dirigée à la peau. Le drainage bi polaire est une voie qui assure le drainage combiné de la cavité résiduelle par des drains placés au sein de cette cavité et de la VBP par un drain de Kehr plus rarement par un drain trans cystique avec ou sans ligature de la fistule kysto-biliaire [4].

La comparaison entre les différentes méthodes chirurgicales dans la littérature montre que la technique de DITFO a donné la plus basse morbidité et le séjour hospitalier le plus court. Par contre, le drainage bi polaire est grevé de la plus lourde morbidité avec le plus long séjour hospitalier (Tableau 6, Tableau 7). Selon les résultats des différentes études dans la littérature, la technique de DITFO représente le traitement de choix du KHF largement ouvert dans les voies biliaires. Elle doit être proposée de première intention, selon l´étude multicentrique tunisienne publiée en 2010 par Zaouch *et al*. [5], en cas de kystes <15cm après résection du dôme saillant, périkyste souple et les kystes centraux qui détruisent la convergence. C´est une technique séduisante qui a donné le plus faible taux de morbi-mortalité avec une morbidité variant de 0 à 16% et une mortalité quasiment nulle et un séjour hospitalier le plus court de 8 à 15 jours. Mais cette méthode ne peut être utilisée que dans 30% des cas [5-10].

**Tableau 6:** comparaison entre les différentes méthodes chirurgicales en fonction de la morbidité spécifique

	DITFO	PERDROMO	DB	DBAL
Zaouch	1,9%	12%	71,2%	41,3%
Daali	-	0	4,7%	20,3%
Moujahed	-	0	57%	16,7%
Baraket	0	0	13,6%	4,5%
Medarhri	-	15,4%	63%	-
Jarrar	2%	4%	16%	6%
Daldoul	16%	-	-	-
Daldoul	-	20%	-	-

DB: drainage bipolaire; DBAL: drainage bipolaire avec ligature; MS: morbidité spécifique; DITFO: drainage interne transfistulo-oddien

**Tableau 7:** comparaison entre les différentes méthodes chirurgicales en fonction de la durée du séjour hospitalier

	DITFO	PERDROMO	DB	DBAL
Zaouch	15	-	-	20
Daali	-	15	27	15
Moujahed	-	28	30	40
Baraket	9,5	20	25	10,5
Medarhri	-	25	30	-
Jarrar	8,7	17,2	23	12
Notre série	11	22	14	12

DB: drainage bipolaire; DBAL: drainage bipolaire avec ligature; DITFO: drainage interne transfistulo-oddien

Le PERDROMO est indiquée en cas de contre-indication au DITFO (kystes >15cm ou périkyste épais qu´on n´arrive pas à assouplir) ou en cas d´impossibilité de la suture directe de la fistule en tissu sain. Cette technique a donné de bons résultats avec une morbidité variant de 0 à 20% et une mortalité quasiment nulle. Cependant, elle nécessite d´une part un long séjour hospitalier pour obtenir l´exclusion de la fistule (supérieure à 25 jours dans la majorité des séries). D´autre part, la méthode de PERDROMO a des complications spécifiques à la technique qui sont en premier lieu la désinsertion de la sonde de cholédochostomie de l´orifice fistuleux qui peut donner un tableau de cholépéritoine et en deuxième lieu le fait que la sonde de cholédochostomie est trop poussée dans le cholédoque obstruant ainsi le drain de Kehr [11].

Le drainage bi polaire avec ligature est indiquée en cas de fistule terminale avec la possibilité de suture en un tissu sain (12,2), et le drainage bi polaire avec respect de la fistule est indiquée si la fistule est sectorielle ou tronculaire et latérale et en cas de non-faisabilité des autres méthodes thérapeutiques (PERDROMO et DITFO). La morbidité du drainage bipolaire avec respect de la fistule varie selon les séries de 4,7 à 63% avec un séjour hospitalier allant de 20 à 30 jours. Cette morbidité élevée et ce long séjour hospitalier jugé le plus long parmi les autres méthodes conservatrices a amené des auteurs à condamner cette technique qui n´est utilisée que par nécessité [5-9]. La mortalité varie de 0 à 0,9%. La morbidité de la déconnexion kysto-biliaire par suture directe de la fistule varie selon les séries de 4,5 à 41,3%. Ces mauvais résultats s´expliquent par une suture dans un tissu fibrosé ou calcifié qui est un facteur prédictif d´échec de cette méthode. La mortalité varie de 0 à 5%.

## Conclusion

L´ouverture du kyste dans les voies biliaires est une complication redoutée du KHF car elle pose des problèmes de réparation chirurgicale. Il n´y a pas de consensus en l´absence d´études prospectives randomisées qui peuvent codifier cette prise en charge. La technique de DITFO représente la technique de choix dans le traitement des FKB, elle est à une morbidité moins élevée. Le PERDROMO est une alternative en cas de contre-indication au DITFO. Le drainage bipolaire associé à une ligature de la fistule peut donner de bons résultats en cas de fistule terminale avec un périkyste souple.

### Etat des connaissances sur le sujet

- La technique de DITFO représente le traitement de choix du KHF largement ouvert dans les voies biliaires. Elle doit être proposée de première intention en cas de kystes <15cm après résection du dôme saillant, périkyste souple et les kystes centraux qui détruisent la convergence;
- Le PERDROMO est indiquée en cas de contre-indication au DITFO ou en cas d´impossibilité de la suture directe de la fistule en tissu sain;
- Le drainage bi polaire avec ligature est indiqué en cas de fistule terminale avec la possibilité de suture en un tissu sain. Cependant, le drainage bi polaire avec respect de la fistule ne doit être utilisé que par nécessité.

### Contribution de notre étude à la connaissance

- Un patient sur 10, présentant un KHF de foie largement ouvert dans les voies biliaires, avait un bilan hépatique strictement normal;
- L´échographie abdominale et le scanner ne permet de visualiser la fistule que dans la moitié des cas.
